# Durable treatment response to Trastuzumab deruxtecan in two patients with recurrent grade III anaplastic ependymoma

**DOI:** 10.1186/s40348-026-00263-y

**Published:** 2026-10-07

**Authors:** Dietmar Krex, Irina A. Kerle, Arne Grey, Stephanie Pleißner, Stephan Richter, Christoph Heining, Stefan Fröhling, Hanno Glimm, Martin Bornhäuser, Gunnar Folprecht, Andy Göbel, Cornelia S. Link-Rachner

**Affiliations:** 1 Department of Neurosurgery, Technische Universität Dresden, Faculty of Medicine and University Hospital Carl Gustav Carus, Fetscherstrasse 74, Dresden, 01307 Germany; 2 Department for Translational Medical Oncology, National Center for Tumor Diseases Dresden (NCT/UCC), a partnership between DKFZ, Faculty of Medicine and University Hospital Carl Gustav Carus, TUD Dresden University of Technology, and Helmholtz-Zentrum Dresden - Rossendorf (HZDR), Dresden, Germany; 3 Translational Medical Oncology, Faculty of Medicine and University Hospital Carl Gustav Carus, TUD Dresden University of Technology, Dresden, Germany; 4 German Cancer Consortium (DKTK), partner site Dresden, a partnership between DKFZ and University Hospital Carl Gustav Carus Dresden, Dresden, Germany; 5 Department of Neuroradiology, Technische Universität Dresden, Faculty of Medicine and University Hospital Carl Gustav Carus, Fetscherstrasse 74, Dresden, 01307 Germany; 6 Department of Medicine 1, Technische Universität Dresden, Faculty of Medicine and University Hospital Carl Gustav Carus, Fetscherstrasse 74, 01307 Dresden, Germany; 7 Division of Translational Medical Oncology, German Cancer Research Center (DKFZ), Heidelberg, Germany; 8 National Center for Tumor Diseases (NCT), NCT Heidelberg, a partnership between DKFZ and Heidelberg University Hospital, Heidelberg, Germany; 9 Division of Translational Precision Medicine, Institute of Human Genetics, Heidelberg University, Heidelberg, Germany; 10 German Cancer Consortium (DKTK), Core Center Heidelberg, Heidelberg, Germany; 11 Department of Medicine 3, Technische Universität Dresden, Faculty of Medicine and University Hospital Carl Gustav Carus, Fetscherstrasse 74, Dresden, 01307 Germany

## Abstract

**Background:**

Anaplastic ependymoma is a rare and aggressive central nervous system malignancy for which effective systemic treatment options are limited. Trastuzumab deruxtecan (T-DXd), an antibody–drug conjugate targeting human epidermal growth factor receptor 2 (HER2), has demonstrated intracranial activity in patients with HER2-positive solid tumors. We report two patients with recurrent, heavily pretreated anaplastic ependymoma and molecular evidence of HER2 activation who received T-DXd as off-label therapy.

**Methods:**

Both patients underwent comprehensive molecular profiling, including whole-genome and transcriptome sequencing and proteomic analyses. HER2 expression was subsequently assessed by immunohistochemistry. Treatment response was evaluated by serial magnetic resonance imaging (MRI), while clinical benefit and treatment-related toxicities were documented longitudinally.

**Results:**

Both patients had multiply recurrent anaplastic ependymoma and had exhausted conventional local and systemic treatment options. Molecular profiling identified elevated ERBB2 expression at the transcriptomic and/or proteomic level, with HER2 immunohistochemical positivity in approximately 80% and 15% of tumor cells, respectively. The first patient received T-DXd and achieved sustained disease stabilization after an initial mixed radiographic response, with ongoing disease control for 26 months. The second patient initially received temozolomide plus lapatinib but experienced progressive disease. Following treatment with T-DXd disease stabilization was achieved and maintained for 14 months at the most recent follow-up. Treatment-related hematologic and hepatic toxicities were manageable with supportive measures, dose modification, and temporary treatment interruption.

**Conclusions:**

These two cases demonstrate durable disease stabilization with T-DXd in heavily pretreated patients with recurrent anaplastic ependymoma and molecular evidence of HER2 activation. The clinical benefit observed, including after failure of lapatinib-based HER2-targeted therapy, supports further investigation of T-DXd in molecularly selected patients with recurrent ependymoma. Comprehensive molecular profiling may help identify patients who could benefit from HER2-directed treatment in this rare and therapeutically challenging disease.

**Supplementary Information:**

The online version contains supplementary material available at https://doi.org/10.1186/s40348-026-00263-y.

## Introduction

Trastuzumab deruxtecan (T-DXd) is an antibody–drug conjugate comprising a humanized monoclonal anti-human epidermal growth factor receptor (HER)2 antibody linked to the topoisomerase I inhibitor deruxtecan [1]. T-DXd is approved for the treatment of advanced HER2-positive breast and gastric cancer [2]. Approximately 15–20% of breast cancer cases are HER2-positive, and up to 50% of patients with advanced HER2-positive disease develop brain metastases during the course of their illness [3].

Although initially considered too large to pass the blood brain barrier, visualisation studies have shown intracranial activity of Cu-tagged trastuzumab [4]. A hypothesized mechanisms is, that the blood brain barrier is disrupted at the site of metastases allowing larger molecules to penetrate the brain parenchyma [5].

Evidence of intracranial activity of T-DXd has been demonstrated in a pooled analysis of the DESTINY-Breast01, DESTINY-Breast02, and DESTINY-Breast03 trials, which reported an intracranial objective response rate of approximately 45% in patients with brain metastases [6]. These results were further confirmed in the phase 3b/4 DESTINY-Breast12 study, providing additional support for the efficacy of T-DXd in this patient population [7]. Based on these findings, T-DXd is currently under investigation for the treatment of primary brain cancers such as glioblastoma (NCT06058988).

Anaplastic ependymomas are rare (0.2–0.4 cases per million annually) but aggressive WHO Grade III brain tumours, typically affecting children or young adults, with the highest proportion occurring in newborns and children up the age of 4 [8]. Depending on location, surgical resection often paired with postoperative radiation therapy remains the primary treatment approach to achieve long-term curation. There is currently no standard systemic therapy for ependymoma. Some patients may profit from the use of temozolomide in combination with lapatinib. However, clinical efficacy remains moderate with median progression-free survival (PFS) of 0.65 years and 1-year PFS of 38% [9].

Considering the lack of established therapeutic options apart from surgery and/or radiation, targeted treatment approaches are an increasingly attractive option for affected patients.

## Case 1

**Fig. 1:**
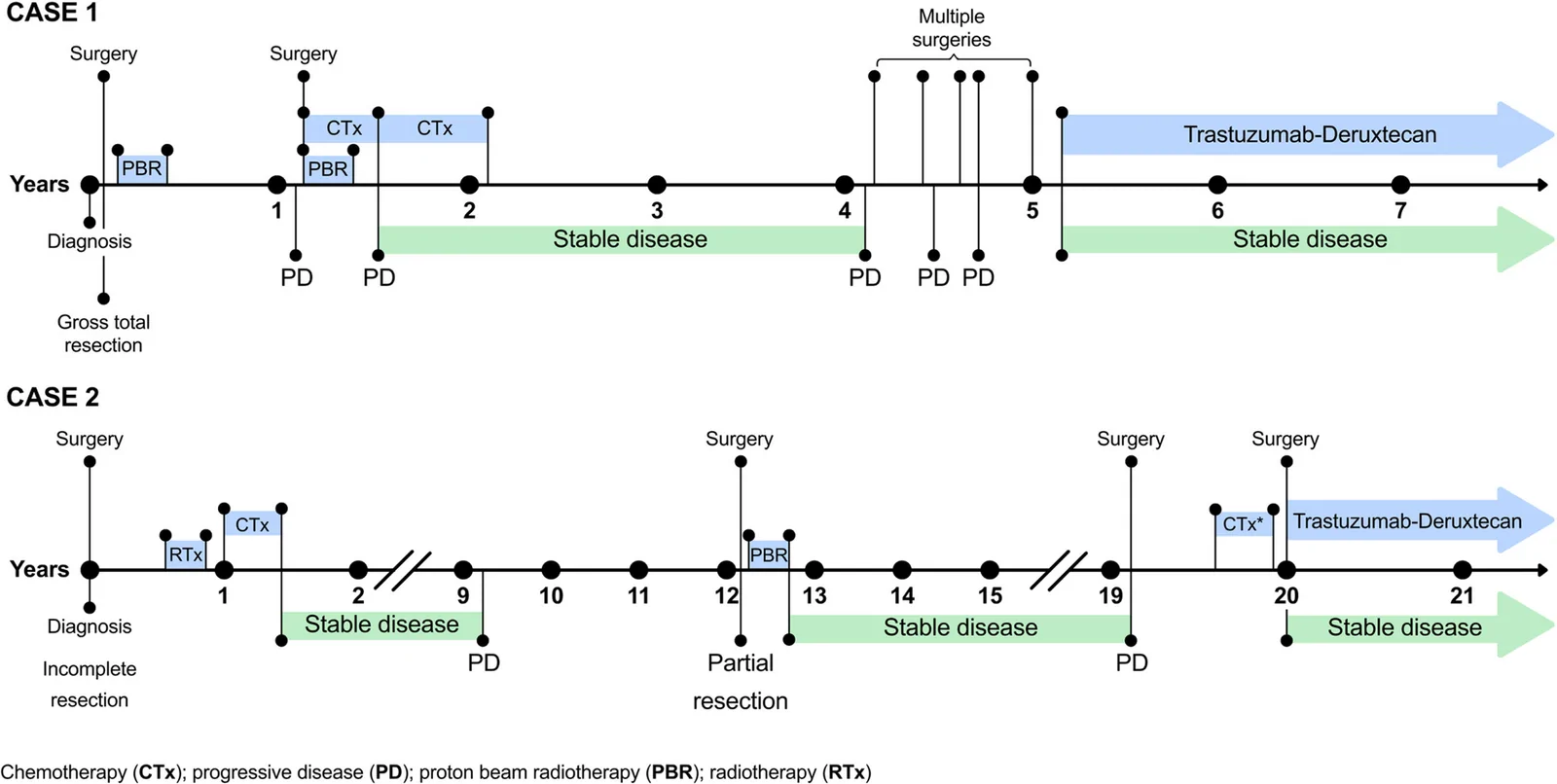
Timeline of 2 patients with anaplastic ependymoma. Abbreviations: CTx, chemotherapy; PD, progressive disease; PBR, proton beam radiotherapy; RTx, radiotherapy

We report the case of a Caucasian female who was initially diagnosed with a supratentorial anaplastic ependymoma at the age of 16 years (Fig. 1). Molecular diagnostic analysis revealed a ZFTA fusion, with no evidence of gene fusions involving YAP1, C11orf95, FGFR1–3, ALK, BRAF, ROS1, or RET. Following gross macroscopic tumor resection, she received proton beam radiotherapy (59.4 Gy). Within 12 months, follow-up magnetic resonance imaging (MRI) revealed relapse at multiple intracranial sites. Subsequent surgery enabled resection of most metastatic lesions and was followed by a second course of proton therapy and chemotherapy with etoposide and trofosfamide.

This intensive multimodal treatment resulted in stable disease with residual tumor remnants. However, approximately two years later, progressive disease was detected. During the following 12 months, rapid disease progression necessitated five additional tumour resections (Fig. 1).

Given the extensive disease progression and the increasing difficulty of achieving local tumour control by surgery, the patient was enrolled in the DKFZ/NCT/DKTK MASTER (Molecularly Aided **S**tratification for Tumor Eradication Research program, ClinicalTrials.gov ID: NCT05852522) program, a precision oncology program for comprehensive molecular characterization by whole-genome (WGS) and transcriptome sequencing (TS), and additional multi-omics analyses such as (phospho-)proteomics [10].

**Fig. 2:**
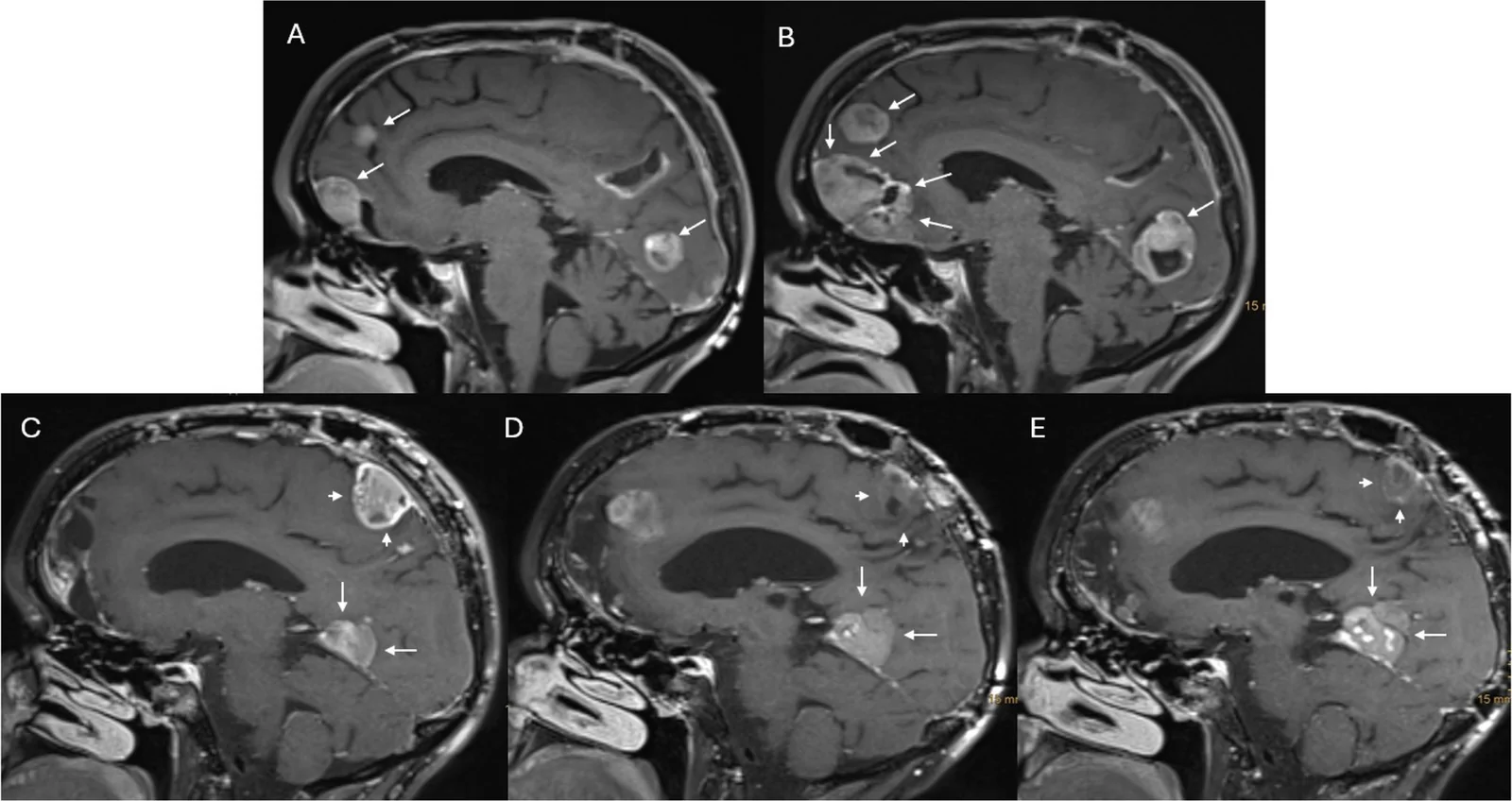
Course of an anaplastic ependymoma before and after the start of treatment with Trastuzumab-Deruxtecan. A 21-year-old female patient with numerous intracranial manifestations of an ependymoma. **A** shows three extra-axial tumor manifestations in the left occipital and frontal regions (arrows). Next follow up MRI, performed only 6 weeks later (**B**) depicts a rapid progression of all lesions. **C** shows the baseline MRI scan immediately before the start of Trastuzumab therapy. On the left parietal region, solid tumor of a recurrent lesion that has undergone surgery (arrow heads). A further lesion is visible on the left occipital region (arrows). Follow up MRIs, performed 19 months (**D**) and 26 months (**E**) later, show regression of the recurrent parietal tumor (arrow heads) and a very slow growth of the solid occipital tumor (arrows). The contrast-enhancing lesion seen frontal in (**D**) and **E** corresponds to reactive enhancement following surgery and does not represent a new tumor manifestation. **A-E**: Axial reconstructions of the 3D-T1-weighted sequence after intravenous contrast application

For proteomic and phosphoproteomic analyses, TMT-based MS3 intensities were normalized and transformed to quasi-LFQ intensities; fold changes and z-scores were calculated relative to the continuously growing pan-cancer background cohort of the MASTER program, with no ependymoma-specific reference range applied. Comprehensive molecular profiling identified a potential therapeutic rationale for HER2-targeted treatment, revealing elevated ERBB2 expression at both the proteomic (fold change 4.21, z-score 1.55) and phosphoproteomic levels (maximum fold change 36, z-score 2). Immunohistochemical analysis subsequently confirmed HER2 expression in approximately 80% of tumor cells (Suppl. table 1, for full data access, check data availability statement at the end of manuscript) Fig. 2.

Based on these findings, off-label treatment with T-DXd was initiated with a starting dose of 5.4 mg per kg body weight every 3 weeks, in accordance with the established breast cancer protocol [2]. In the seventh treatment cycle, the dose was increased once to 6.4 mg per kilogram of body weight in accordance with the established protocol for gastric cancer. This was done in an attempt to obtain an even better tumor response. However, this higher dose was not maintained due to increased and prolonged nausea as well as earlier onset and longer-lasting fatigue and continued at regular dose (5.4 mg per kg body weight). After 39 cycles, the dose of T-DXd had to be temporarily halted due to liver toxicity and was later resumed at a reduced dose.

During the first six months of treatment, MRI assessments demonstrated a mixed radiographic response. Thereafter, disease stabilization was achieved and has been maintained on serial MRI evaluations. This radiological benefit was accompanied by a marked clinical improvement as reported by the patient, allowing her to actively participate in family life and social activities such as vacations. The patient currently undergoes MRI surveillance every three months and has maintained stable disease for 26 months since the initiation of T-DXd therapy.

## Case 2

The second case involved a young Caucasian female who was diagnosed with an infratentorial anaplastic ependymoma at 4 years of age. Subsequent molecular assessment using the Illumina Human Methylation 935 Array yielded a classifier score of 0.92, consistent with posterior fossa ependymoma. Copy-number variation (CNV) profiling demonstrated a gain of chromosome arm 1q. The MGMT promoter was unmethylated. Based on the methylation profile and immunophenotype, the tumor was classified as posterior fossa ependymoma, group A (PFA), with loss of H3K27me3 expression.

Initial treatment consisted of partial surgical resection, chemotherapy according to the HIT-SKK 2000 protocol (three cycles), radiotherapy with a total dose of 54 Gy, and maintenance chemotherapy according to the HIT 2000 protocol (four blocks).

Approximately nine years after the initial diagnosis, routine MRI surveillance revealed a small tumour recurrence in the right cerebellopontine angle. Subsequent MRI examinations demonstrated slow but continuous growth of the lesion, and surgical resection was recommended. However, the patient and her family initially opted for a watch-and-wait approach. Following further tumour growth, a second surgical procedure was performed three years later, approximately 12 years after the initial diagnosis. Histopathological examination confirmed recurrent ependymoma. As complete resection was not feasible, proton beam radiotherapy (54 Gy) was administered, resulting in a reduction of the residual lesion.

Because residual tumour tissue, adjuvant chemotherapy with oral temozolomide was recommended. However, this treatment was declined by the patient and her family, and the patient continued with radiographic surveillance. At that time, persistent neurological deficits included central facial nerve palsy and impaired hearing on the right side.

Following these treatments, stable disease was maintained for additional six years. Thereafter, MRI surveillance demonstrated progression of the residual tumour. Given the extensive prior treatment history, the patient was enrolled in the precision oncology MASTER program.

Whole-genome and transcriptome sequencing revealed ERBB2 mRNA overexpression and elevated expression at the proteomic level (Suppl. table 1, for full data access, check data availability statement at the end of manuscript). In addition, HER2 immunohistochemistry was performed and demonstrated HER2 expression in approximately 15% of tumour cells. Based on these findings, an off-label treatment with temozolomide and lapatinib, a targeted HER2/EGFR inhibitor, was initiated. At the first follow-up assessment, imaging showed a slight increase in the size of the known ependymoma. Nevertheless, the interdisciplinary tumour board recommended continuation of therapy. Unfortunately, subsequent short-term follow-up imaging confirmed further tumour progression.

Alternative treatment options were therefore considered. Due to the lack of remaining radiation reserve, re-irradiation was not feasible, and complete surgical resection was likewise impossible. Based on the favourable experience observed in patient 1, an alternative off-label treatment approach with T-DXd (5.4 mg per kg body weight every 3 weeks) was initiated following another partial tumour resection. Of note, due to prolonged hematologic toxicity with neutropenia within the first cycles, pegfilgrastim is now routinely administered 24 h after application of T-DXd in order to maintain the regular treatment cycles without dose reduction.

This treatment is well tolerated and at the most recent follow-up, 14 months after initiation of T-DXd, the patient demonstrates stable disease. Given the rapid tumour progression observed during prior treatment with temozolomide and lapatinib, this outcome has been considered a clinically meaningful therapeutic success.

## Discussion

Anaplastic ependymomas are rare central nervous system tumours for which evidence-based systemic treatment options remain limited due to the low incidence of the disease and the consequent lack of adequately powered clinical trials.

In such rare tumour entities, comprehensive molecular profiling can provide valuable therapeutic opportunities by identifying actionable molecular alterations and enabling the implementation of targeted treatment strategies. In the presented cases, disease control could not be achieved in the two young female patients despite extensive multimodal treatment, including proton beam radiotherapy, systemic chemotherapy, and multiple surgical resections. In both cases, tumour progression accelerated markedly during the year preceding the initiation of T-DXd, highlighting the urgent need for alternative therapeutic approaches.

The patients were subsequently enrolled in the MASTER program, a precision oncology initiative integrating comprehensive molecular analyses to guide individualized treatment decisions [10]. Molecular profiling revealed pronounced ERBB2 expression in both patients.

While the first patient was treated directly with T-DXd, the second patient initially received temozolomide in combination with lapatinib. However, no radiographic response was observed, and short-interval MRI follow-up examinations demonstrated continued disease progression. Treatment was therefore switched to T-DXd, resulting in a favourable clinical and radiographic response, as outlined above. Although some hemato- and hepatotoxicity was observed in both patients, these were manageable using supportive measures or dose adaptations and the clinical benefit experienced by both patients outweighed adverse effects significantly.

T-DXd is currently approved in HER2-positive breast cancer (including HER2-low/ultralow subgroups), gastric/gastroesophageal tumours and non-small cell lung cancer. The durable disease stabilization observed following initiation of T-DXd suggests that HER2-directed therapy may represent a promising treatment strategy in selected patients with recurrent ependymoma harbouring evidence of HER2 pathway activation. This is especially remarkable in patient 2, where lapatinib failed to provide clinical benefit.

Although HER2 overexpression has been described in subsets of ependymomas, clinical evidence supporting HER2-targeted therapies in this disease remains scarce. Our findings therefore add to the emerging evidence that molecularly guided treatment approaches may provide meaningful clinical benefit in heavily pretreated patients with recurrent ependymoma.

## Supplementary Information

Supplementary Material 1.

## Data Availability

The data generated in this study are available via controlled access in the German Human Genome-Phenome Archive (GHGA, data.ghga.de) under the GHGA Accession https://data.ghga.de/study/GHGAS80221339908861. Further details, including the data access policy for the study, can be found there.
