# Supplementary material for: Durable treatment response to Trastuzumab deruxtecan in two patients with recurrent grade III anaplastic ependymoma
Source: Mol Cell Pediatr. 2026 Oct 7;13:50. doi: 10.1186/s40348-026-00263-y (PMC13646213; doi:10.1186/s40348-026-00263-y)
Supplement: Supplementary file 1 [file 40348_2026_263_MOESM1_ESM.docx]

**Table 1:** Results/recommendations from molecular tumour board

**Case #1:**

| **Recommendation** | **Rationale** | **LoE** | **Priority** |
| --- | --- | --- | --- |
| ERBB2-targeted therapy, e.g., with trastuzumab deruxtecan (off-label),  possibly in combination | ERBB2 elevated protein expression (rank 34, FC 4.21, z-score 1.55; below significance threshold) and significant phosphoproteomic overexpression (maximum FC 36, z-score 2). | m2A | 1 |
| SSTR5-targeted therapy, e.g., with pasireotide or lutathera (off-label),  possibly as part of a combination therapy | SSTR5 mRNA overexpression  (rank 2, 374.6-fold, FPKM 15.47) | m2B | 2 |
| PARP inhibition, e.g., with olaparib (off-label), possibly as part of a combination therapy | Mutation signature SBS3 (29%) | m2B | 3 |
| NOTCH1-targeted therapy with brontictuzumab, in future studies | NOTCH1 mRNA overexpression (rank 4, 4.1-fold, FPKM 36.07) | m2C | 4 |
| BCL-targeted therapies, such as navitoclax, in future studies | ASCL1 mRNA overexpression (rank 4, 811.1-fold, FPKM 25.19) | m3 | 5 |

**Case #2:**

| **Recommendation** | **Rationale** | **LoE** | **Priority** |
| --- | --- | --- | --- |
| ERBB2-targeted therapy, e.g., with lapatinib and temozolomide | ERBB2 mRNA expression (rank 23, 3.5-fold, FPKM 35.95)  ERBB2 elevated protein expression (rank 84, FC 3.23, z-score 1.14; below significance threshold)  ERBB4 mRNA expression (rank 34, 5.7-fold, FPKM 0.29)  HER2 immunohistochemistry 1+ | m3 | 1 |
| PARP inhibition, as part of a combination therapy | Mutation signature SBS3 (37%) | m2B | 2 |
| CDK4/6 inhibitor, as part of a combination therapy | CDK6: Gain (TCN 2.99/2.00P) | m2C | 3 |

LoE: Level of evidence. Molecular evidence levels according to the classiﬁcation scheme developed for the MTBs of the National Center for Tumor Diseases (NCT) and within the German Cancer Consortium (DKTK): Leichsenring, J. et al. Variant classification in precision oncology. Int. J. Cancer 145, 2996–3010 (2019)

FPKM: Fragments Per Kilobase of transcript per Million mapped reads

SBS: Single-base substitution. SBS classification according to <https://cancer.sanger.ac.uk/signatures/sbs>

Proteomic analysis: TMT-based MS3 intensities were normalized and transformed to quasi-LFQ intensities. Protein and phosphopeptide intensities were log10-transformed, and fold changes and z-scores were calculated relative to the continuously growing pan-cancer background cohort established within the DKFZ/NCT/DKTK precision-oncology programs, including the MASTER cohort. No ependymoma-specific reference range was used. The methodology is described in detail by Schneider et al. (preprint: <https://www.biorxiv.org/content/10.64898/2026.07.08.737171v1>).
